# A new species of *Monoliropus* Mayer, 1903 (Crustacea, Amphipoda, Caprellidae) from Korean waters

**DOI:** 10.3897/zookeys.517.9915

**Published:** 2015-08-13

**Authors:** Soon-Sang Hong, Jun-Haeng Heo, Young-Hyo Kim

**Affiliations:** 1 Department of Life Sciences, Dankook University, Cheonan, Korea 330-714

**Keywords:** *Monoliropus*, Caprellidae, Amphipoda, new species, key, Korea

## Abstract

A new species of the genus *Monoliropus* belonging to the family Caprellidae was collected from the Yellow Sea, Korea. The new species differs from *Monoliropus agilis* Mayer, 1903, *Monoliropus kazemii* Momtazi & Sari, 2013, and *Triprotella amica* Arimoto, 1970 as follows: 1) gnathopod 1 subrectangular; 2) pereonites 2–3 with acute triangular processes anterolaterally; 3) mandibular palp, apical article with four simple setae subdistally. The new species is fully illustrated and extensively compared with related species. This is the first record of the genus *Monoliropus* from Korean waters. A key to *Monoliropus* species is also given.

## Introduction

The genus *Monoliropus* Mayer, 1903 is one of 57 genera belonging to the family Caprellidae. *Monoliropus* is closely related to *Metaprotella* Mayer, 1890 and *Triprotella* Arimoto, 1970 and commonly characterized by having biarticulate flagellum of antenna 2; triarticulate mandibular palp; pereonites 3–4 with gills; uniarticulate pereopods 3–4; well developed, 6-articulate pereopod 5; in male, abdomen with a pair of biarticulate appendages and a pair of lobes (Arimoto 1976). To date, this genus *Monoliropus* is comprised of seven described species (Momtazi and Sari 2013; WoRMS 2015). In this article, a full description of the new species in the genus *Monoliropus* from Korean waters is provided, with a brief description of the female, focusing on the sexually dimorphic characters. This is the first record of the genus *Monoliropus* from Korea and a key to the world *Monoliropus* species is also provided.

## Material and methods

**Figure 1.**
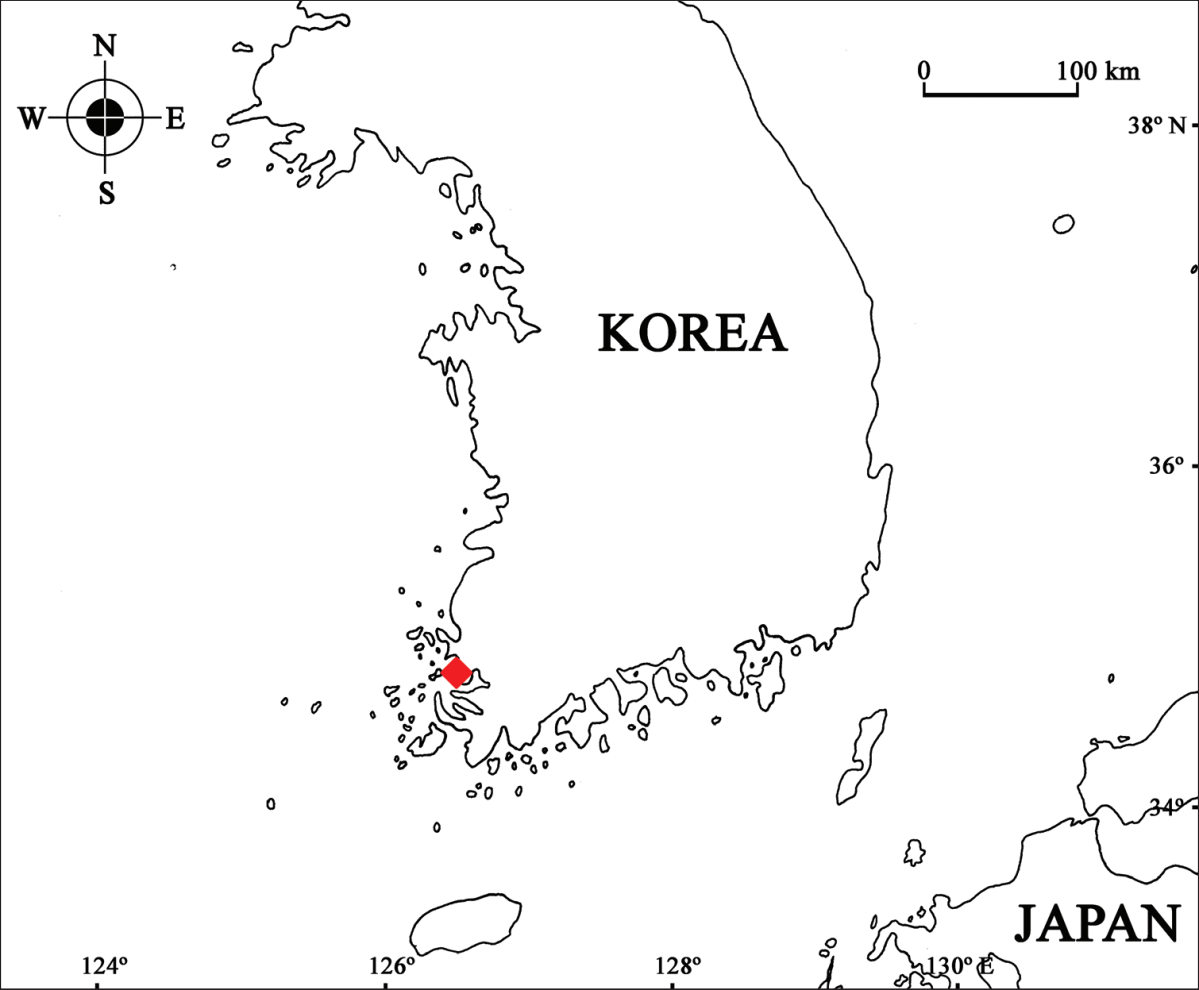
Distribution of *Monoliropus leeae* sp. n. from Korean waters (♦: Bukhang Port, Jukgyo-dong, Mokpo-si, Jeollanam-do, Korea).

Specimens were collected by light trap from the subtidal zone of Bukahng Port, Mokpo-si, Korea in 2012 (Fig. 1). The specimens were fixed with 80% ethanol and dissected in glycerin on Cobb’s aluminum hollow slides. Drawings and measurements were performed with the aid of a drawing tube, under a stereomicroscope (Olympus SZX12; Tokyo, Japan) and a differential interference contrast microscope with Nomarski optics (Olympus BX51). Type specimens were deposited at the National Institute of Biological Resources (NIBR), Incheon, Korea and the Department of Life Sciences, Dankook University (DKU), Cheonan, Korea.

## Taxonomy

### Genus Monoliropus

Taxon classificationAnimaliaAmphipodaCaprellidae

Mayer, 1903

Korean name: Jjal-eun-a-ga-mi-da-ri-ba-da-dae-beol-rae-sok, new

#### Type species.

*Monoliropus agilis* Mayer, 1903

#### Diagnosis.

Antenna 2, flagellum biarticulate, swimming setae absent; mandibular palp bi- or triarticulate; pereonites 3–4 with gills; pereopods 3–4 present, uniarticulate; in male, abdomen with a pair of uni- or biarticulate appendages and a pair of lobes.

#### Species composition.

The genus contains seven species, *Monoliropus agilis* Mayer, 1903, *Monoliropus concavimanus* Horton, 2008, *Monoliropus enodis* Rayol & Serejo, 2003, *Monoliropus falcimanus* Mayer, 1904, *Monoliropus hapipandi* Guerra-García, 2004, *Monoliropus kazemii* Momtazi & Sari, 2013, and *Monoliropus tener* Arimoto, 1968.

### Monoliropus leeae sp. n.

Taxon classificationAnimaliaAmphipodaCaprellidae

http://zoobank.org/4D5A1B2A-1B84-402A-B5E4-397084742D53

Korean name: Jjal-eun-a-ga-mi-da-ri-ba-da-dae-beol-rae, new

**Figure 2.**
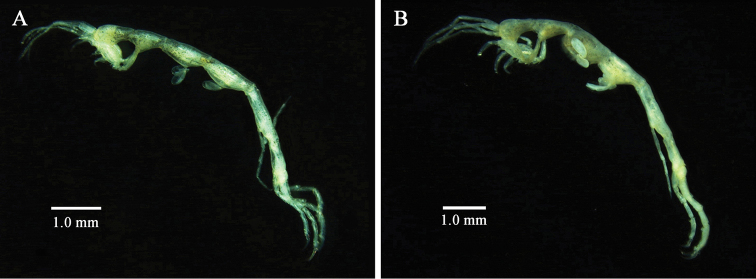
*Monoliropus leeae* sp. n. **A** male, 5.6 mm **B** immature female, 6.4 mm.

**Figure 3.**
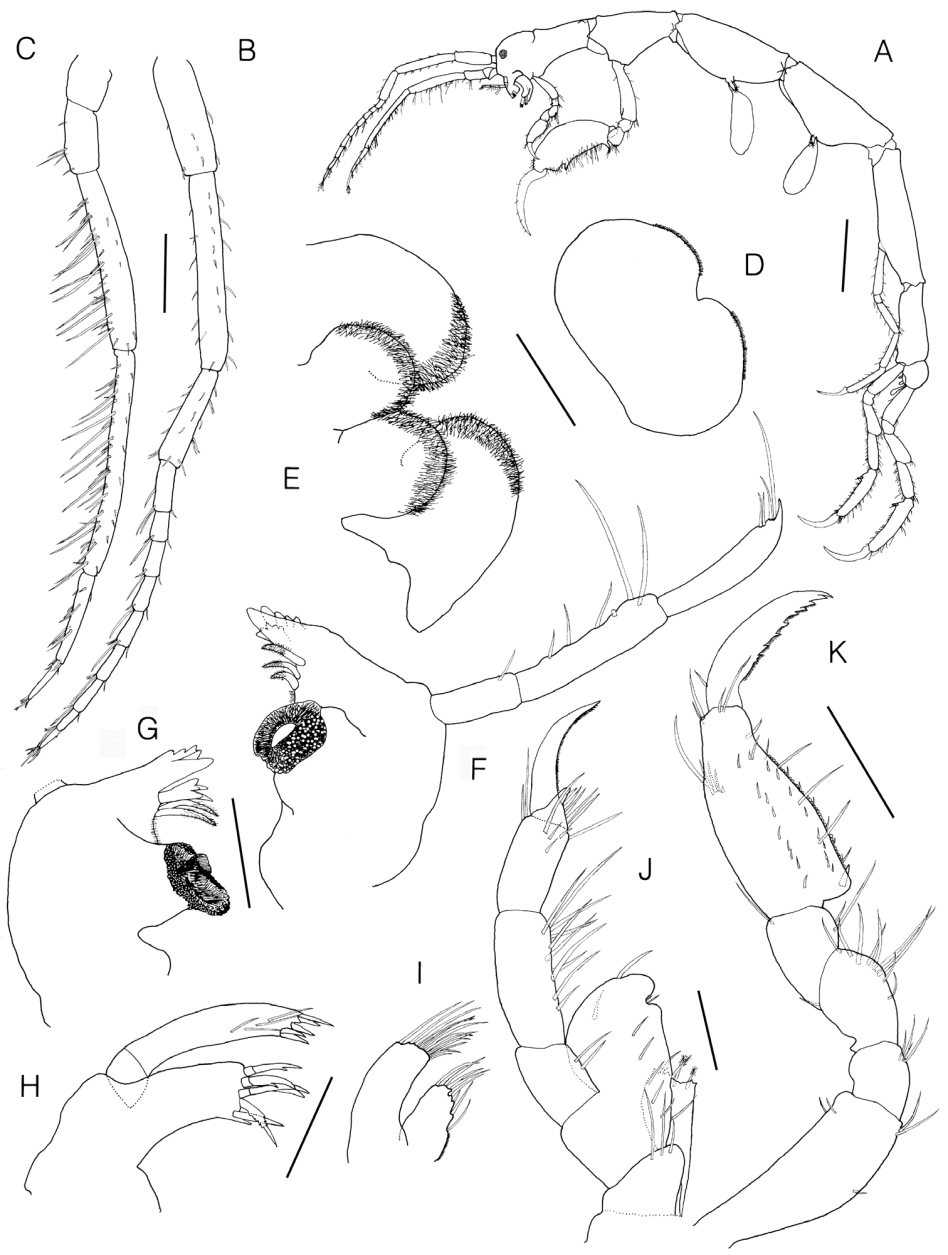
*Monoliropus leeae* sp. n., holotype, male, 9.3 mm. **A** habitus, lateral view **B** Antenna 1 **C** Antenna 2 **D** upper lip **E** lower lip **F** left mandible **G** right mandible **H** maxilla 1 **I** maxilla 2 **J** right maxillied **K** gnathopod 1. Scale bars: 1.0 mm (**A**), 0.3 mm (**B, C**), 0.2 mm (**K**), 0.1 mm (**D–J**).

**Figure 4.**
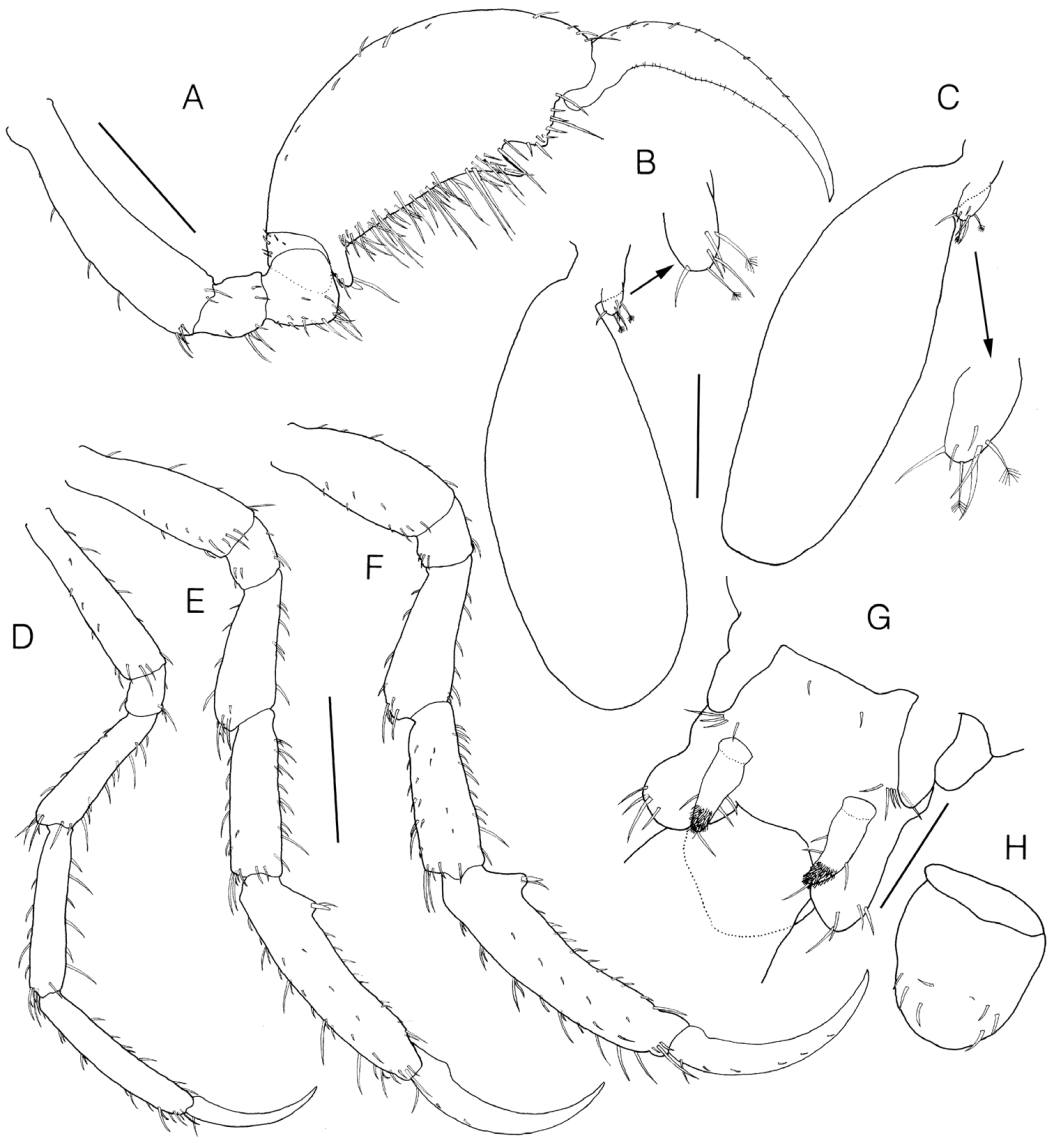
*Monoliropus leeae* sp. n., holotype, male, 9.3 mm. **A** gnathopod 2 **B** gill 3 and pereopod 3 **C** gill 4 and pereopod 4 **D** pereopod 5 **E** pereopod 6 **F** pereopod 7 **G** abdomen, ventral view **H** single dorsal lobe, dorsal view. Scale bars: 0.4 mm (**A, D–F**), 0.2 mm (**B, C**), 0.1 mm (**G, H**).

**Figure 5.**
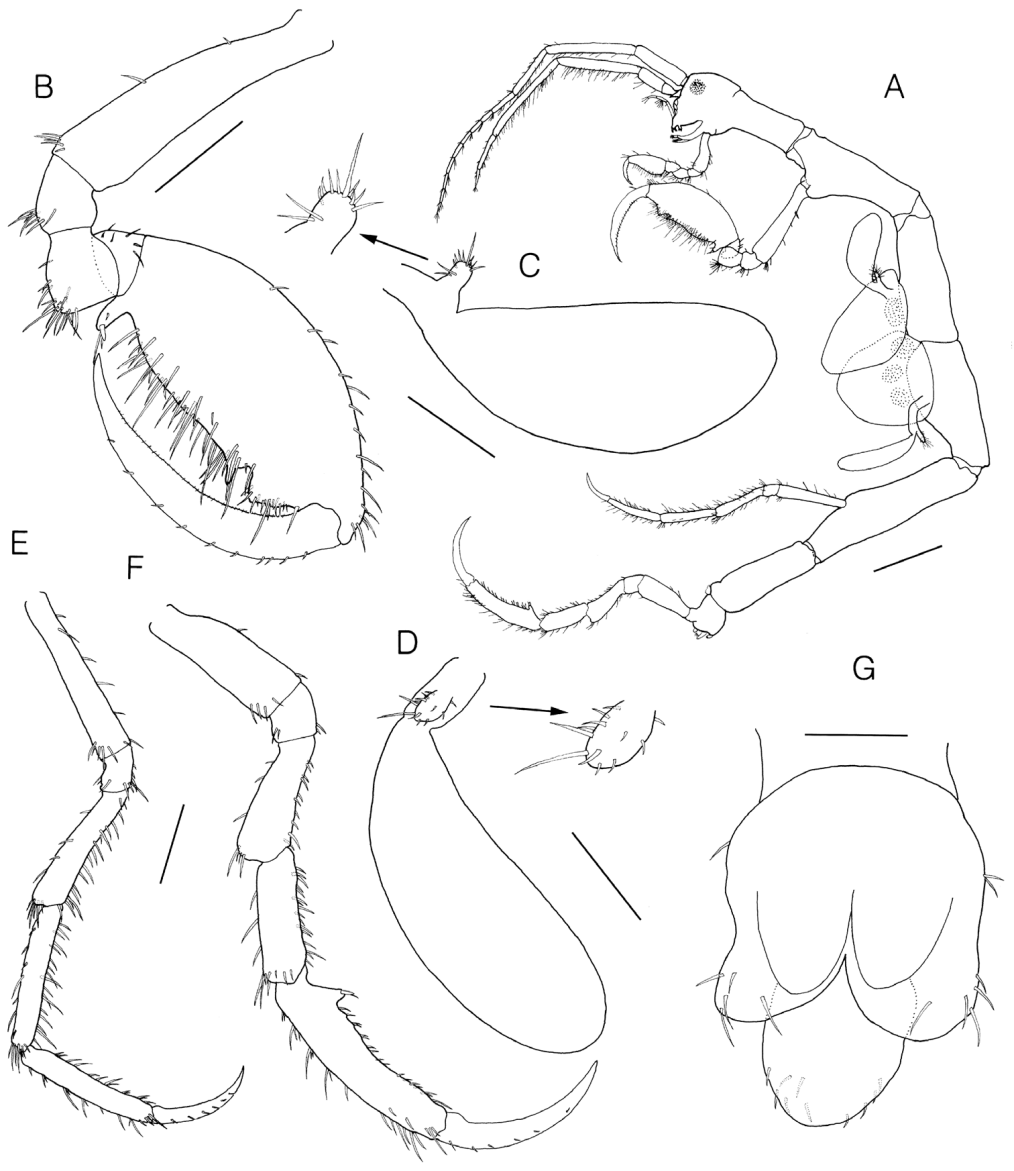
*Monoliropus leeae* sp. n., paratype, female, 11.7 mm. **A** habitus, lateral view **B** gnathopod 2 **C** gill 3 and pereopod 3 **D** gill 4 and pereopod 4 **E** pereopod 5 **F** pereopod 6 **G** abdomen, ventral view. Scale bars: 1.0 mm (**A**), 0.4 mm (**B**) 0.3 mm (**E, F**), 0.2 mm (**C, D**), 0.1 mm (**G**).

Figures 1 , 2 , 3 , 4 , 5

#### Type material.

Holotype: male, 9.3 mm, NIBRIV0000309619, Bukhang Port, Jukgyo-dong, Mokpo-si, Jeollanam-do, Korea, 34°48'00"N, 126°21'56"E, S.S. Hong and S.H. Kim, by light trap from 6–8 m depth, 11 July 2012. Paratypes: female, 11.7 mm, NIBRIV0000309620, 27 July 2012, same station data as holotype; 6 males, 5.3–6.7 mm, DKUAMP201501, 11 July 2012, same station data as holotype; 2 immature males and 5 immature females, 5.3–7.1 mm, DKUAMP201502, 27 July 2012, same station data as holotype.

#### Description.

**Holotype**, **male**, NIBRIV0000309619.

Body (Fig. 3A) 9.3 mm long, slender and long, surface smooth. Head round and smooth without projection. Eye small, round. Head and pereonite 1 fused, suture present. Pereonite 1 nearly smooth, with 1 small bump anterodorsally and a pair of minute blunt processes posterodorsally. Pereonite 2 with acute triangular process anterolaterally. Pereonites 3–4 subequal in length, with small uniarticulate pereopods and rounded gills ventrally, and tiny triangular process on both lateral sides. Pereonite 5 subrectangular, width 0.30 × length, with 6-articulate pereopod. Pereonite 6 smooth without process. Length ratio of pereonites 1–7 = 1.00 : 1.52 : 2.12 : 2.23 : 2.53 : 1.40 : 0.49.

Antenna 1 (Fig. 3B) 0.35 × body; length ratio of peduncular articles 1–3 = 1.00 : 3.46 : 0.84; flagellum 9-articulate, 0.76 × peduncular articles, each article with 1 or 2 aesthetascs ventrodistally.

Antenna 2 (Fig. 3C) slightly shorter than antenna 1; length ratio of peduncular articles 3–5 = 1.00 : 2.75 : 3.45; peduncular articles 4–5 with unequal simple setae ventrally; flagellum biarticulate, 0.30 × peduncular articles, proximal article elongate, 1.95 × distal article.

Upper lip (Fig. 3D) rounded, notched midventrally with pubescence apically.

Lower lip (Fig. 3E) well developed, inner and outer lobes with patch of pubescence apically.

Left mandible (Fig. 3F) incisor and lacinia mobilis 5-teethed; setal row with 3 pectinated setae; molar well developed, truncate; mandibular palp slender, triarticulate, length ratio of articles 1–3 = 1.00 : 1.92 : 1.85, article 2 with 6 simple setae, distal article acute apically, with 4 simple setae.

Right mandible (Fig. 3G) similar to left except setal row with 2 pectinated setae and molar flake present.

Maxilla 1 (Fig. 3H) inner plate absent; outer plate with 6 stout setal teeth (3 simple, 2 bifid and 1 denticulate) apically; palp biarticulate, distal article with 5 apical spines and 4 subapical setae.

Maxilla 2 (Fig. 3I) inner plate with dense pubescence medially and 8 simple setae on apical and subapical margins; outer plate longer than inner, with 12 simple setae apically.

Maxilliped (Fig. 3J) inner plate subrectangular, with 1 forked and 3 penicillate setae apically; outer plate much larger than inner plate, distomedial portion with rounded groove, distal margin rounded with 1 simple seta; palp 4-articulate, article 3 with subacute process apically, distal article falcate, with a row of setules along inner margin, length ratio of articles 1–4 = 1.00 : 1.75 : 1.70 : 1.17.

Gnathopod 1 (Fig. 3K) propodus subrectangular, narrowing distally, width 0.45 × length, palm serrated with 1 proximal grasping spine; dactylus falcate, with irregular serrations on inner margin; length ratio of 6 articles = 1.00 : 0.26 : 0.38 : 0.34 : 1.02 : 0.77.

Gnathopod 2 (Fig. 4A) anterior margin of carpus very short; propodus massive, width 0.44 × length, anterior margin convex, with rounded angle, palmar margin straight with proximal blunt process bearing grasping spine and acute poison tooth followed by rounded notch subdistally; dactylus elongate, falcate; length ratio of 6 articles = 1.00 : 0.25 : 0.27 : 0.18 : 1.34 : 1.10.

Gill 3 (Fig. 4B) elongate, elliptical, 0.63 × pereonite 3.

Pereopod 3 (Fig. 4B) vestigial, uniarticulate, 0.07 × gill, with 4 simple and 2 penicillate setae.

Pereopod 4 (Fig. 4C) similar to pereopod 3, but slightly longer.

Pereopod 5 (Fig. 4D) well developed, slender, setose, 6-articulate, 1.21 × pereonite 5, inserted about 7/10 from the anterior end of pereonite 5; length ratio of 6 articles = 1.00 : 0.21 : 0.74 : 0.87 : 0.94 : 0.68.

Pereopod 6 (Fig. 4E) well developed, setose, 6-articulate, 2.49 × pereonite 6, 1.20 × pereopod 5, attached to the posterodistal end of the pereonite 6; propodus subrectangular, palm defined by posterodistal blunt bump with grasping spine and seta; length ratio of 6 articles = 1.00 : 0.28 : 0.76 : 0.84 : 1.40 : 1.00.

Pereopod 7 (Fig. 4F) similar and subequal to pereopod 6, length ratio of 6 articles = 1.00 : 0.26 : 0.78 : 0.90 : 1.50 : 1.10.

Penes (Fig. 4G) cylindrical in shape, situated medially, width 0.50 × length.

Abdomen (Fig. 4G, H) with a pair of appendages, a pair of lateral and single dorsal lobes; appendage uniarticulate, with 3 lateral, 1 apical setae, distal portion covered with patch of fine setules; lateral lobe with 4 simple setae apically; dorsal lobe rounded, with 7 simple setae dorsally.

**Paratype**, **female** (sexually dimorphic characters), NIBRIV0000309620.

Body (Fig. 5A) 11.7 mm long. Body form generally as in male including antennae 1–2, but pereonites 3–4 with rounded brood pouches. Gnathopod 2 (Fig. 5B) palm slightly curved convexly. Pereopods 3–7 (Fig. 5C–F) more setose than male. Abdomen (Fig. 5G) lacking appendages, lateral lobe wider than that of male.

#### Remarks.

The genus *Monoliropus* belongs to the family Caprellidae, which has close affinities with *Metaprotella* Mayer, 1890 and *Triprotella* Arimoto, 1970 as it possesses the following characters: 1) antenna 2, peduncles without swimming seta, flagellum biarticulate; 2) mandibular palp bi- or triarticulate; 3) pereopods 3–4 reduced, uniarticulate; 4) in male, abdomen with uni- or biarticulate appendages. However, *Metaprotella* is distinguished from *Monoliropus* by setal formula 1–x–y–1 of the distal article of mandibular palp and fused pereonites 6–7. *Triprotella* is very similar to the genus *Monoliropus*; however, is discernible from *Monoliropus* by setal formula 1-1-1 of the distal article of the mandibular palp, uniarticulate pereopods 3–4, morphology of the gnathopod 2, and form of abdomen (Sivaprakasam 1977; Laubitz 1991; Guerra-García 2002). The species *Monoliropus agilis* has been redescribed by Guerra-García (2004) , showing a setal formula of mandibular palp of 1-1-1, and the abdomen very similar to that of the genus *Triprotella*. Therefore both genera could be re-established or synonymized in the future. The new species *Monoliropus leeae* sp. n. is similar to *Monoliropus agilis* Mayer, 1903, *Monoliropus kazemii* Momtazi & Sari, 2013, and *Triprotella amica* Arimoto, 1970, however, is distinguished from its congeners based on the characters listed in Table 1 and the combination of the following features: 1) body medium sized, 9–11 mm (*vs.* small sized, 4 mm in *Monoliropus agilis*, 5–7 mm in *Triprotella amica*); 2) maxilla 1, outer plate with six stout setal teeth (*vs.* five in *Triprotella amica*, seven in *Monoliropus agilis* and *Monoliropus kazemii*); 3) mandibular palp, distal article with four simple setae (*vs.* three simple setae in *Monoliropus agilis* and *Triprotella amica*); 4) gnathopod 1, propodus subrectangular (*vs.* subtriangular in *Monoliropus agilis*, *Monoliropus kazemii*, and *Triprotella amica*); 5) gnathopod 1, dactylus with serrations on inner margin (*vs.* with serrations both margins in *Triprotella amica*); 6) pereonites 2–3 with acute triangular processes anterolaterally (*vs.* without triangular processes in *Monoliropus agilis*, *Monoliropus kazemii*, and *Triprotella amica*); 7) pereopods 3–4 short (*vs.* elongate in *Monoliropus agilis*, *Monoliropus kazemii*, and *Triprotella amica*); 8) abdominal appendage uniarticulate (*vs.* biarticulate in *Monoliropus kazemii*).

**Table 1.** Morphological characters of *Monoliropus leeae* sp. n. and closely related species.

Characters	Species (male)				
	*Monoliropus agilis*	*Monoliropus kazemii*	*Triprotella amica*	*Triprotella amica*	*Monoliropus leeae* sp. n.
Body length (mm)	4.0	8.5	5.4	6.3	9.3
Pereonites 2–3, anterior processes	×	○	no referred	×	○
Right mandible, molar flake	○	×	no referred	○	○
Mandibular palp, distal article, # of setae	3	4	3	3	4
Maxilla 1, outer plate, # of setae	7	7	no referred	5 setae	6 setae
Maxilliped, outer plate, distal margin	jagged	jagged	jagged	jagged	rounded
Gnathopod 1, propodus	subtriangular, width 0.61 × length	subtriangular, width 0.49 × length	no referred	subtriangular, width 0.80 × length	subrectangular, width 0.45 × length
Gnathopod 1, dactylus serrations	inner margin	inner margin	no referred	both margins	inner margin
Pereopods 3–4	elongate, 2.7–2.9 × width	elongate, 3.6–3.7 × width	elongate	elongate, 3.5 × width	short, 1.3–1.9 × width
Abdominal appendage, # of setae	uniarticulate, no seta	biarticulate, 5 setae	uniarticulate, 1 seta	uniarticulate, 1 seta	uniarticulate, 4 setae
Abdomen, dorsal lobe, # of setae	2 terminal setae	2 terminal setae	no referred	2 terminal setae	7 dorsal setae
Distribution	Phuket, Thailand (Guerra-García 2004)	Persian and Oman Gulf, Iran (Momtazi and Sari 2013)	Arabian Sea, Oman (Arimoto 1970)	Mbudya island, Tanzania (Guerra-García 2002)	Mokpo-si, Korea (Present study)

#### Etymology.

The specific name *leeae* is in honor of Dr. Kyung-Sook Lee, who has contributed to knowledge of Korean caprellid Amphipoda.

#### Distribution.

Bukhang Port, Jukgyo-dong, Mokpo-si, Jeollanam-do, Korea.

### Key to the species of *Monoliropus* (in male)

**Table:** 

1	Mandibular palp 2-articulate, with 1 single seta distally	***Monoliropus hapipandi* Guerra-García, 2004**
–	Mandibular palp 3-articulate, with several setae distally	**2**
2	Gnathopod 2, propodus without grasping spine and process on palmar margin	***Monoliropus tener* Arimoto, 1968**
–	Gnathopod 2, propodus with grasping spine and process on palmar margin	**3**
3	Pereopod 5, propodus with grasping spine	**4**
–	Pereopod 5, propodus without grasping spine	**5**
4	Gnathopod 2, propodus, palmar margin straight	***Monoliropus kazemii* Momtazi & Sari, 2013**
–	Gnathopod 2, propodus, palmar margin concave	***Monoliropus falcimanus* Mayer, 1904**
5	Abdominal appendage biarticulate	***Monoliropus enodis* Rayol & Serejo, 2003**
–	Abdominal appendage uniarticulate	**6**
6	Gnathopod 2, propodus, palmar margin concave	***Monoliropus concavimanus* Horton, 2008**
–	Gnathopod 2, propodus, palmar margin straight	**7**
7	Gnothopod 1, propodus subtriangular; pereopods 3–4 elongate, length > 2.5 × width	***Monoliropus agilis* Mayer, 1903**
–	Gnothopod 1, propodus subrectangular; pereopods 3–4 short, length < 2.0 × width	***Monoliropus leeae* sp. n.**

## Supplementary Material

XML Treatment for Monoliropus

XML Treatment for Monoliropus leeae
